# The Effect of Micro-Computed Tomography Thresholding Methods on Bone Micromorphometric Analysis

**DOI:** 10.3390/jfb15110343

**Published:** 2024-11-13

**Authors:** Arda Buyuksungur, Bence Tamás Szabó, Adrienn Dobai, Kaan Orhan

**Affiliations:** 1 Department of Basic Medical Sciences, Ankara University, 06100 Ankara, Türkiye; 2 Department of Oral Diagnostics, Faculty of Dentistry, Semmelweis University, 1088 Budapest, Hungarydobai.adrienn.gyongyi@semmelweis.hu (A.D.); knorhan@dentistry.ankara.edu.tr (K.O.); 3 Department of Dentomaxillofacial Radiology, Ankara University, 06100 Ankara, Türkiye

**Keywords:** bone micromorphometry, micro CT, connectivity, bone volume, trabecular separation

## Abstract

Bone micromorphometric parameters are generally analyzed with micro CT to reveal two- and three-dimensional structures. These parameters are generally used for new bone formation studies such as tissue engineering and biomaterials studies. Different threshold methods are used for the image segmentation of bone micromorphometric parameters. However, these different threshold methods provide different results for the bones analyzed. This study aimed to compare thresholding methods to evaluate bone micromorphometric parameters in the mouse bone. A dataset containing 15 mouse tibia was used to analyze the different thresholding methods for bone micromorphometric parameter analysis. These threshold methods were used to analyze the mouse tibia (*n* = 15) with thresholded bones. The threshold methods and the analysis were used directly from CTAn (Bruker Micro-CT). The results were compared between the threshold methods, which included bone volume, trabecular number, connectivity, trabecular separation, and other parameters. There was agreement to some extent for all bone micromorphometric analyses using the different thresholding methods. The results showed that the thresholding method showed good agreement for connectivity and trabecular thickness, but the other parameters showed limited agreement. The evaluation of threshold methods allows for the comparison of image segmentation and the quantification of mouse tibia micromorphometric parameters. This study may enable the analysis of bone micromorphometric parameters using the relatively close threshold method in image segmentation across different research groups.

## 1. Introduction

Imaging bones is an essential part of the investigation of bone structure. Micro CT is a gold standard for investigating bone microstructures and scanning bones at high resolution in vitro [1]. After acquisition, the resulting radiographs are reconstructed to create images of the scanned bones in three planes.

The segmentation process is one of the main parts of image analysis. The process is based on separating images into two or more homogeneous segments, and one of the most common methods is thresholding [2]. To assess the morphology of the bone, analyses carried out on reconstructed images are prone to thresholding. Thresholding is the method of image segmentation that creates binary images. Before many types of micro CT analysis, binarization was carried out, which is the transformation of a grey image into a black and white image, and it is very difficult to standardize [3]. The threshold usually uses the image’s histogram and categorizes the pixels into different groups [2]. There are three types of thresholding: global, adaptive, and local. The entire image dataset is processed based on a single threshold of an image in case of global thresholding. Still, unique threshold values are used for partitioned sub-images obtained from the whole image used in local thresholding [4]. The adaptive thresholding of each pixel in the image is calculated.

The different phases in micro CT images are segmented via a grey value threshold in the greyscale histogram of the input scan [5]. In 8-bit images, the grey values are between 0–255 (black–white). According to the threshold, selected grey values are assigned to a new value, white and black [1]. The threshold is used to separate air from the materials and the materials from each other, and it is based on the intrinsic characteristics of a material [6,7]. Its use can allow for the differentiation of the newly formed bone from the ex-bone structure, trabecular bone from cortical bone, or biomaterials from the bone and other tissues. The threshold value detects the background and the objects so that if it is lower than the threshold value, it is calculated as the background, and if it is higher than the value, it is calculated as an object [8]. If the composite material is scanned with micro CT, the threshold is used to differentiate the materials from each other and analyze the inner structure properties. The choice of threshold is very important because it will change the results in 2D and 3D, such as the fracture network expanded or contracted according to the choice of the threshold [9]. If the analysis is carried out above the optimum threshold, the results will be overestimated, and if it is below the optimum value, then the results will be underestimated. Therefore, the choice directly affects the results of the materials. One of the motivations of this manuscript is to detect the comparison of the thresholding method of choice and how bone micromorphometric results are affected using different algorithm-based thresholding methods.

Although these selections are inconsistent, global thresholding is generally used for bone studies [10]. Some bone studies use two different thresholds, bone and non-peak, using the greyscale histogram [11]. It is also recommended that the visual control of the segmentation with the greyscale images is necessary to separate the objects from each other [12].

Although global thresholding can be used for segmentation, it relies on operator interpretation [13]. Operator-dependent segmentation introduces certain uncertainty to the results, which can be very sensitive when the pore sizes are very small [14]. A local adaptive threshold is generally used for complex objects; the optimal threshold can change throughout the object [14]. Global thresholding defines a single threshold for the object [7]. The effectiveness of the automated thresholding can change according to the dataset type and the application [7,15,16,17,18,19]. Micro CT is widely used in bone micromorphometric analysis, and the image analysis’s thresholding is the main part. AI-based systems can be used in different image segmentation studies, but until this manuscript was written, no AI-based systems had worked on micro CT data. It is certain that, in a few years, AI-based systems of thresholding methods will be used in micro CT data. Knowledge about different thresholding methods would allow for a better interpretation of the results and the differences between the studies using micro CT.

Therefore, this study’s main objective was to evaluate the available thresholding algorithms of CTAn software (v1.19, Bruker Micro CT, Kontich, Belgium) and their effects on bone morphometric analysis. The results of this study will help guide future micro CT studies that use different thresholding methods.

## 2. Materials and Methods

### 2.1. Micro-Computed Tomography (μCT) Analysis

**Figure 1:**
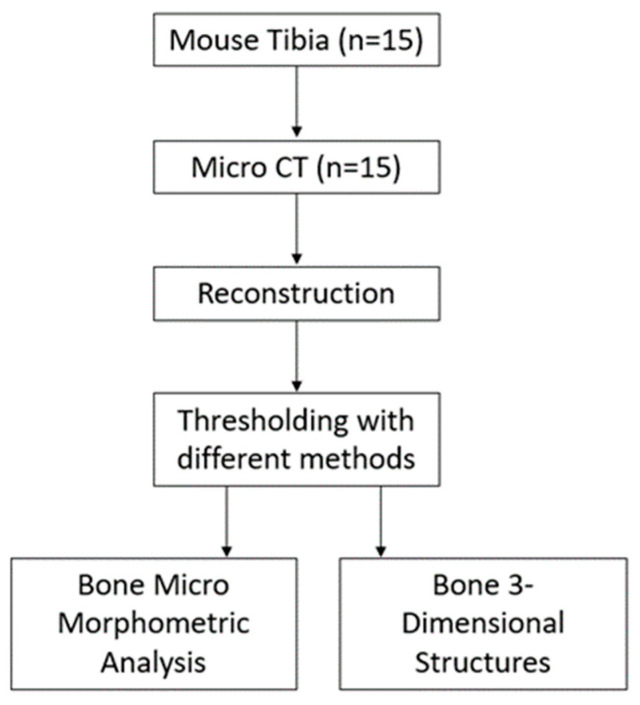
Flowchart of the study groups and procedures.

The mouse (C57BL/6) tibia (n = 15) was scanned with a micro CT device (1172, Bruker micro CT, Kontich, Belgium) using a 5 um voxel size. The samples were scanned at a voltage of 70 kV and a current of 124 mA using a 484 ms exposure time and a 0.5° rotation step. For image noise reduction, an Al 0.5 mm aluminum filter was used. Figure 1 shows the experimental design according to the analysis carried out to compare the thresholding methods.

Figure 1 explains the experimental procedures for bone micromorphometric analysis. Scanning NRecon software (v.1.7.4.6., Bruker micro CT, Kontich, Belgium) was used to reconstruct each raw image sequence of the bone samples according to the manufacturer’s recommendations. The reconstruction parameters were smoothing = 2, ring artifact reduction = 5, and beam hardening correction = 45%. Two blinded examiners performed the quantitative analysis (A. B. and B. T. S). The examiners carried out the analysis and the results were obtained. The examiners did not assign any score for the results; the results were represented quantitatively. CTVox was used for volume rendering to investigate the sample in 3D, and CTVol was used to visualize the 3D model formats.

### 2.2. Analysis of Bone

The reconstructed images were imported to CTAn (v1.19., Bruker Micro CT, Kontich, Belgium) for further analysis. Regions of interest (ROIs) were selected in the diaphyseal site with 400 slices for trabecular bone and about 500 slices from the growth plate level. Different thresholding methods were applied to determine tissue volume (TV), bone volume (BV), bone surface (BS), bone surface/volume ratio (BS/BV), percent bone volume (BV/TV), connectivity (Conn.), trabecular number (Tb.N), trabecular thickness (Tb.Th), and trabecular separation (Tb.Sp) micromorphometric parameters. The definitions of the relevant micromorphometric variables measured in this study are listed in Table 1.

### 2.3. Threshold Analysis

This study is focused on different thresholding methods to detect the differences between the results. Thresholding is used to segment the data collected from scanning and is a selection for the grey of the material. The threshold methods were used to create binarized images of bones. Therefore, 11 different thresholding methods were applied for each bone sample to determine the differences between the micromorphometric values. The thresholding methods used for the analysis are global, Adaptive Median-C, Adaptive Mean-C, Adaptive Mid-range C, Automatic Otsu, Two-dimensional (Otsu), Automatic Ridler–Calvard, Automatic Quantile, Automatic Mean, Automatic Mid-range, and Automatic Triangle methods. Two independent examiners performed the analysis.

### 2.4. Statistical Analysis

The minimum sample size required for the study was determined as at least 13 samples in each group, with a test power of 80% at a confidence level of 95%, f = 0.35* for One-Way Analysis of Variance, and a Type I error probability of α = 0.05. The relevant sample size calculations were made with the G*power 3.1V program. The analyses were carried out using 15 samples.

Statistical difference was evaluated using the first ANOVA analysis and the Games–Howel post-hoc test. The means between the subgroups analyzed with ANOVA and data are expressed as the mean ± SD. If significant differences were detected between the means, a pairwise comparison between the means was performed with the Games–Howel post-hoc test. Throughout this study, the statistical significance presented *p* < 0.05.

## 3. Results

Global thresholding is a generally used method for image segmentation but depends on operator selection, and determining the best threshold value remains a challenge. The different thresholding methods were compared, and the significant differences are presented.

### 3.1. Threshold Methods

The threshold settings and the properties of the thresholding are summarized in Table 2. The low and high threshold levels are almost identical for all of the methods. All threshold methods and the analysis were carried out using images obtained from scanning with micro CT. After scanning, the reconstruction was carried out, and the same parameters were used for the reconstruction.

Table 2 shows that the threshold methods generally used the upper level of threshold of 255, but the lower threshold changed in the methods.

### 3.2. Optimization of the Threshold

The operators chose the optimal (low–high) global threshold value for the samples. The other threshold methods decide the threshold levels by the method used.

### 3.3. Micromorphometry Analysis

**Figure 2:**
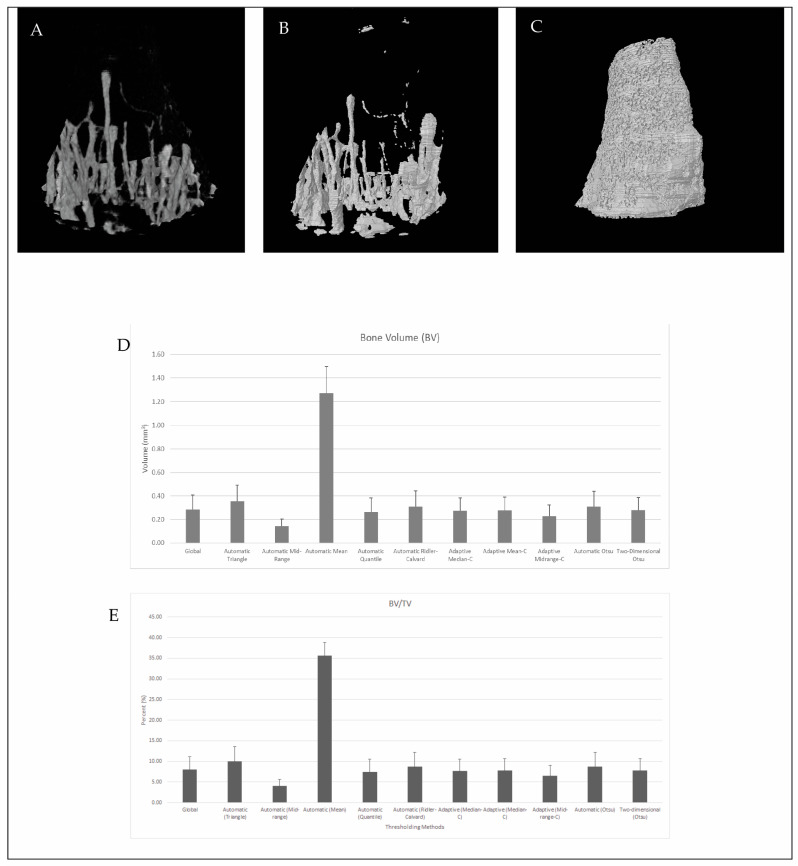
Representative µCT images of the mouse tibia. (**A**) Volume rendering figure of the sample. (**B**) STL model of the same sample after applying the global thresholding method. (**C**) STL model of the same sample after applying the Automatic (Mean) thresholding method. (**D**) A significant comparison between thresholding methods is shown based on BV data. (**E**) A significant comparison between thresholding methods is shown based on BV/TV data.

Micromorphometric data of the mouse tibia were compared after thresholding with CTAn software (Bruker, Kontich, Belgium). The 11 thresholding methods were compared using 15 different mice bones with the settings detailed in Table 2 regarding the 11 different results, which were the most used analysis for the bone microstructure. After thresholding was performed with CTAn, the analyses of each bone were calculated using the same ROI and VOI for each bone, and results were obtained. The analysis showed that there were differences between some parameters and some thresholding methods. The TV values were not significantly different from each other in the results obtained using different thresholding methods, but the BV was significantly higher in Automatic (Mean) thresholding methods. The BV value and the BV/TV found with Automatic (Mean) were significantly higher than those of the other groups (Figure 2A–E).

BV reflects the bone volume in the selected VOI, which is directly determined by the threshold. As shown in Figure 2B,C, the volume rendering image (CTvox, v. 3.3.1 Bruker Micro CT, Kontich, Belgium) almost had the same proportion as the global thresholding method, but the Automatic (Mean) threshold method used expanded the model because the global thresholding used greyscale levels of 80–255, and the Automatic (Mean) method used greyscale levels of 26–255. The model created based on the global thresholding method had 300 × 10^3^ facets in the models, but the model created using the Automatic (Mean) method had 7.3 × 10^6^ facets in the model. The increase in the low threshold level from 26 to 80 (Sample 1) dramatically increased the volume of the bone in the selected VOI.

**Figure 3:**
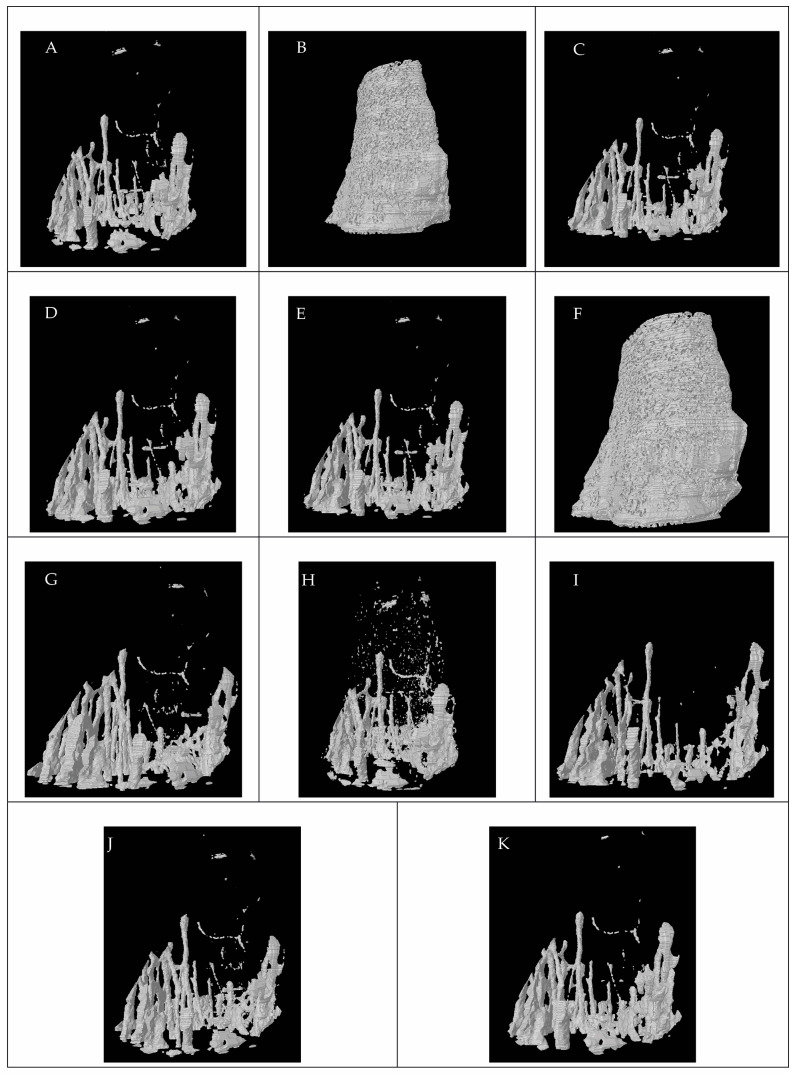
Representative model images of the mouse tibia. (**A**) Global threshold, (**B**) Automatic Mean, (**C**) Adaptive Median (**C**,**D**) Adaptive Mean (**C**,**E**) Adaptive Midrange (**C**,**F**) Automatic Quantile, (**G**) Automatic Ridler–Calvard, (**H**) Automatic Triangle, (**I**) Automatic Midrange, (**J**) Automatic Otsu, (**K**) and Two-dimensional (Otsu).

Figure 3 shows all of the models produced using all of the threshold methods. The models were produced with CTAn software after the thresholding methods selected the grey value. CTVol (v. 2.3.2.0, Bruker Micro CT, Kontich, Belgium) software was used to visualize the models.

Figure 3 shows the images of models produced from all threshold methods. The models reveal that the threshold methods dramatically change the model volume and micromorphometric results. Bone micromorphometric results were affected by the application of different threshold methods. As the methods changed the grey value, the results were affected. The choice of thresholding method significantly affects the structure and the morphology of bone. As in Figure 3, some structural connections were broken, and some structures did not exist. The parameters, including trabecular pattern, trabecular separation, structural model index, and trabecular number, were more sensitive to the thresholding method used. The other analysis was found to be sensitive to the threshold method used but showed that the results were also affected by the threshold method to some extent.

All of the micromorphological analyses were carried out using different thresholding methods, and the results were compared with the statistical analysis. The results from the different thresholding methods are compared within the same micromorphological analysis. Table 3 shows the representative results for the BS/BV and BV/TV. To simplify all results, the tables are presented with the letters. The letters show the pairwise comparison between the results. The letters indicate significant differences between the results pairwise (the statistical significance presented *p* < 0.05). 

Table 3 shows the percent bone volume (BV/TV) and the bone surface/volume ratio bone micromorphometric parameters. For the percent bone volume (BV/TV), there was no significant difference between Global, Automatic Triangle, Automatic Quantile, Automatic Ridler–Calvard, Adaptive Median-C, Adaptive Mean-C, Adaptive Midrange-C, Automatic Otsu, and Two-dimensional (Otsu) methods, but there was a significant difference (*p* < 0.05) between Automatic Mean and the other methods. Also, there was a significant difference (*p* < 0.05) between Automatic Midrange and the other methods used, except for the Adaptive Midrange-C method. For the bone surface/volume ratio (BS/BV), there was also a significant difference (*p* < 0.05) between the Automatic Mean and the other methods (*p* < 0.05). Also, there was a significant difference between the Automatic Midrange and Automatic Triangle (*p* < 0.05). Table 4 shows the results for Trabecular thickness and trabecular separation.

In the Tb.Th (trabecular thickness) bone micromorphometric parameter, there was no significant difference between the Global, Automatic Triangle, Automatic Quantile, Automatic Ridler–Calvard, Automatic Otsu, and Two-dimensional (Otsu) methods. Also, there was no significant difference between the Automatic Midrange, Automatic Mean, Adaptive Median-C, Adaptive Mean-C, and Adaptive Midrange-C methods. However, there was a significant difference between these two groups.

The trabecular separation (Tb.Sp) value showed no significant difference between Global, Automatic Midrange, Automatic Quantile, Adaptive Median-C, Adaptive Mean-C, and Adaptive Midrange-C, but Automatic Triangle, Automatic Mean, Automatic Ridler–Calvard, and Automatic Otsu showed significant differences (*p* < 0.05) from the other methods. Table 5 shows the results of the trabecular number and the trabecular pattern factor bone micromorphometric parameters.

In the Tb.N (trabecular number) bone micromorphometric parameter, the Global, Automatic Triangle, Automatic Quantile, Automatic Ridler–Calvard, Adaptive Median-C, Adaptive Mean-C, Adaptive Midrange-C, Automatic Otsu, and Two-dimensional (Otsu) methods showed no significant difference. The Automatic Mean showed a significant difference from all other methods. Automatic Midrange showed significant differences from Automatic Triangle and Adaptive Mean-C.

The Tb.Pf (trabecular pattern factor) bone micromorphometric method showed very different results regarding the threshold method used. The Global, Automatic Midrange, Automatic Quantile, Automatic Ridler–Calvard, Adaptive Mean-C, Adaptive Midrange-C, Automatic Otsu, and Two-dimensional (Otsu) methods were significantly different from Adaptive Median-C, Automatic Mean, and Automatic Triangle methods. Also, Automatic Triangle and Adaptive Median-C were significantly different from each other, and both were significantly different from the other methods. Table 6 shows the connectivity and structural model index results for the different threshold methods.

In the Conn. (connectivity) bone micromorphometric parameter, the Automatic Mean parameter was significantly different from the other methods except for the Two-dimensional (Otsu) method.

In the SMI (structural model index) bone micromorphometric parameter, the Global, Automatic Ridler–Calvard, and Automatic Otsu methods were significantly different from the other methods. Automatic Triangle was significantly different from all other methods. Automatic Midrange was similar to Automatic Quantile, Adaptive Mean-C, Automatic Mean, Adaptive Median-C, and Adaptive Midrange.

## 4. Discussion

This study examined bone micromorphometric analyses to evaluate thresholding methods using micro CT analysis software, CTAn. Using different threshold methods to determine the bone micromorphometric parameters allowed us to compare both image results (STLs) and quantitative results. The methods estimated in this study are used in different studies [20,21,22,23], but a clear indication for using one over another has not been provided. The present study’s results suggest that using some thresholding methods gives very different bone micromorphometric analysis results. The results show that the thresholding method affected the bone morphometric analyses. The main disadvantage of micro CT is considered to be non-standardization.

The presented study shows the changes in micromorphometric parameters caused by the thresholding method used. The other parameters, such as scanning conditions and reconstruction parameters, affect the bone micromorphometric analyses [2,3,24], but the image processing (threshold) also has a huge effect on the parameters that are investigated. The Otsu threshold and the porosity-adaptive threshold were compared for gas diffusion layers directly with models, and the porosity-adaptive threshold method was found to be not accurate for these studies [25]. Another study used the adaptive method over the global threshold for dental material analysis using micro CT [26]. Several studies are using AI-dependent thresholding segmentation for both medical CT and CBCT data [27,28]. One study used a convolutional neural network (CNN) for the thresholding method and compared it with Otsu thresholding with micro CT data, but it was specific to materials used and directly calculated from the 3D scans, not reconstructed data [29]. Specifically, the Automated Mean threshold method used in this study showed that the images analyzed will lead to less accurate bone micromorphometric parameters. The automated mean threshold method was almost the only method that showed significant differences for all of the parameters. The results also showed that the expert-defined results and the other methods differed in comparing some of the bone micromorphometric parameters. The global threshold method is dependent on the expert selection of the value. Although the global threshold method was in good agreement with the other method, different users may select similar values, but they may not be the same value.

The trabecular thickness and connectivity were in better agreement when compared to the other parameters. These two parameters were the parameters least affected by the threshold method.

The trabecular pattern factor and structure model index parameters were in the worst agreement with the other parameters. These two parameters are directly affected by the threshold method used.

The results obtained confirm that the bone micromorphometric parameters are directly affected by the threshold method used. The main differences were obtained from the trabecular pattern factor and the structural model index parameters. The change in the threshold directly affects binarization, leads to changes in the image, and also affects the bone micromorphometric results.

## 5. Conclusions

Threshold-based segmentation is widely used for image segmentation and image analysis. The selection of the thresholding method used influences bone micromorphometric results. This study presents the effectiveness of the different thresholding techniques through the analysis of bone micromorphometrics. The automated mean method was found to be the most variable method among all methods. The trabecular thickness and connectivity were the parameters that were least affected by the thresholding method used.

The results obtained in this study show that the selection of the threshold method is crucial for the bone micromorphometric parameters, and testing the thresholding methods for the most affected parameters can minimize the degree of sensitivity.

## Figures and Tables

**Table 1:** The descriptions of micromorphometric values investigated in this study using CTAn software [12].

Nomenclature	Description	Unit
TV	Tissue volume: The 3D volume measurement in the selected volume of interest (VOI)	mm^3^
BV	Bone volume: The 3D volume of binarized objects within the VOI	mm^3^
BS	Bone surface: The surface area measured in 3D of all of the solid objects within the VOI	mm^2^
BS/BV	Bone surface/volume ratio: 3D measured ratio of surface to the volume within the VOI	mm^−1^
BV/TV	Percent bone volume: Binarized solid objects in proportion to total VOI	%
Conn.	Connectivity: The degree to which the object’s parts are interconnected multiple times	-
Tb.N	Trabecular number: The number of traversals that a random linear path through the volume of interest makes across a solid structure per unit length.	mm^−1^
Tb.Th	Trabecular thickness: Mean thickness of trabeculae	mm
Tb.Sp	Trabecular separation: Mean distance between trabeculae by binarization within the VOI.	mm
Tb.Pf	Trabecular pattern factor: Comparison of volume and surface in 3D within the VOI.	mm^−1^
SMI	Structural model index: Indication of rods’ and plates’ prevalence in 3D	-

ROI: region of interest; VOI: volume of interest.

**Table 2:** The settings for the thresholding methods used.

Threshold Methods	Settings (Lower and Upper Threshold, Pre-Threshold, Background Information, Methods of Calculation in 2D or 3D, Image Processing Inside VOI, and Radius Information)
Global	Output to: Image, Lower grey threshold: 80, Upper grey threshold: 255
Adaptive Median-C	2D space, Output to Image, Kernel: Round, Radius: 10, Constant: 0, Background: Dark, Pre-threshold: on, Lower grey threshold: 80, Upper grey threshold: 255
Adaptive Midrange-C	Output to: Image, Kernel: Round, Radius: 10, Constant: 0, Background: Dark, Pre-threshold: on, Lower grey threshold: 80, Upper grey threshold: 255
Adaptive Median-C	3D space, Output to Image, Kernel: Square, Radius: 1, Constant: 0, Background: Dark, Pre-threshold: on, Lower grey threshold: 80, Upper grey threshold: 255
Automatic Mean	3D space, Inside VOI, Output to Image, Background: Dark, Lower grey threshold: 26–47, Upper grey threshold: 255
Automatic Ridler–Calvard	3D space, Inside VOI, Output to Image, Background: Dark, Lower grey threshold: 75, Upper grey threshold: 255
Automatic Mid-range	3D space, Inside VOI, Output to Image, Background: Dark, Lower grey threshold: 127, Upper grey threshold: 255
Automatic Otsu	3D space, Inside VOI, Output to Image, Background: Dark, Lower grey threshold: 80, Upper grey threshold: 255
Automatic Quantile	2D space, inside ROI, Output to Image, Background: Dark, Quantile: 0.50, Lower grey threshold: 24–30, Upper grey threshold: 255
Automatic Triangle	3D space, Inside VOI, Output to Image, Background: Dark, Lower grey threshold: 63, Upper grey threshold: 255
Two-dimensional Otsu	3D space, Inside VOI, Output to Image, Kernel: Round, Radius: 1, Background: Dark

**Table 3:** Results of thresholding methods for BS/BV (bone surface/volume ratio) and BV/TV (percent bone volume).

Threshold Method	BS/BV					Threshold Method	BV/TV				
	Mean	Std. Dev.	Pairwise Comparison				Mean	Std. Dev.	Pairwise Comparison		
Global	0.08	0.01	A	B		Global	8.05	3.13	A		
Automatic Triangle	0.08	0.01	A			Automatic Triangle	10.07	3.49	A		
Automatic Midrange	0.1	0.02		B		Automatic Midrange	4.07	1.57		B	
Automatic Mean	0.13	0.01			C	Automatic Mean	35.66	3.22			C
Automatic Quantile	0.08	0.01	A	B		Automatic Quantile	7.4	3.18	A		
Automatic Ridler–Calvard	0.08	0.01	A	B		Automatic Ridler–Calvard	8.74	3.42	A		
Adaptive Median-C	0.09	0.01	A	B		Adaptive Median-C	7.65	2.87	A		
Adaptive Mean-C	0.08	0.01	A	B		Adaptive Mean-C	7.76	2.95	A		
Adaptive Midrange-C	0.09	0.01	A	B		Adaptive Midrange-C	6.49	2.48	A	B	
Automatic Otsu	0.08	0.01	A	B		Automatic Otsu	8.73	3.41	A		
Two-dimensional (Otsu)	0.08	0.01	A	B		Two-dimensional (Otsu)	7.85	2.82	A		

**Table 4:** Results of thresholding methods for Tb.Th (trabecular thickness) and Tb.Sp (Trabecular Separation).

Threshold Method	Tb.Th				Threshold Method	Tb.Sp					
	Mean	Std. Dev.	Pairwise Comparison			Mean	Std. Dev.	Pairwise Comparison			
Global	57.12	5.52	A		Global	360.84	115.87	A	B		
Automatic Triangle	59.1	5.64	A		Automatic Triangle	165.44	18.18			C	
Automatic Midrange	43.73	6.35		B	Automatic Midrange	477.27	141.56	A			
Automatic Mean	42.78	8.21		B	Automatic Mean	39.12	6.2				D
Automatic Quantile	55.37	5.93	A		Automatic Quantile	393.36	125.71	A	B		
Automatic Ridler–Calvard	58.15	4.97	A		Automatic Ridler–Calvard	306.23	108.04		B		
Adaptive Median-C	45.18	2.98		B	Adaptive Median-C	359.58	116.15	A	B		
Adaptive Mean-C	48.39	3.44		B	Adaptive Mean-C	359.98	116	A	B		
Adaptive Midrange-C	47.85	4.55		B	Adaptive Midrange-C	360.95	115.3	A	B		
Automatic Otsu	58.12	4.94	A		Automatic Otsu	307.32	107.84		B		
Two-dimensional (Otsu)	57.33	3.57	A		Two-dimensional (Otsu)	374.86	106.57	A	B		

**Table 5:** Results of thresholding methods for Tb.Pf (trabecular pattern factor) and Tb.N (trabecular number).

Threshold Method	Tb.Pf							Threshold Method	Tb.N				
	Mean	Std. Dev.	Pairwise Comparison						Mean	Std. Dev.	Pairwise Comparison		
Global	0.04	0.01	A		C			Global	0.00141	0.00054	A	B	
Automatic Triangle	0.06	0.02				D		Automatic Triangle	0.0017	0.00057	A		
Automatic Midrange	0.04	0.01	A	B				Automatic Midrange	0.00094	0.00034		B	
Automatic Mean	0.05	0.01		B		D	E	Automatic Mean	0.00872	0.00226			C
Automatic Quantile	0.04	0.01	A		C			Automatic Quantile	0.00133	0.00054	A	B	
Automatic Ridler–Calvard	0.04	0.01	A				E	Automatic Ridler–Calvard	0.00151	0.00063	A	B	
Adaptive Median-C	0.03	0.01			C			Adaptive Median-C	0.00171	0.00069	A		
Adaptive Mean-C	0.03	0.01	A					Adaptive Mean-C	0.00162	0.00064	A	B	
Adaptive Midrange-C	0.04	0.01	A				E	Adaptive Midrange-C	0.00137	0.00055	A	B	
Automatic Otsu	0.04	0.01	A		C		E	Automatic Otsu	0.00151	0.00063	A	B	
Two-dimensional (Otsu)	0.03	0.01	A		C			Two-dimensional (Otsu)	0.00137	0.00051	A	B	

**Table 6:** Results of thresholding methods for Conn. (connectivity) and SMI (structural model index).

**Threshold Method**	**Conn.**						
	**Mean**	**Std. Dev.**	**Pairwise Comparison**				
Global	806.80	481.33	A				
Automatic Triangle	1188.53	514.61	A				
Automatic Midrange	669.07	492.58	A				
Automatic Mean	32,318.00	15,244.29			B		
Automatic Quantile	749.67	470,74	A				
Automatic Ridler–Calvard	960.67	778.77	A				
Adaptive Median-C	1224.53	674.83	A				
Adaptive Mean-C	948.13	540.73	A				
Adaptive Midrange-C	885.93	568.93	A				
Automatic Otsu	958.87	779.05	A				
Two-dimensional (Otsu)	668.47	311.73	A		B		
**Threshold Method**	**Structure model index**						
	**Mean**	**Std. Dev.**	**Pairwise Comparison**				
Global	2.77	0.21	A				
Automatic Triangle	4.35	0.70				D	
Automatic Midrange	2.43	0.18		B	C		E
Automatic Mean	2.37	0.80	A		C		
Automatic Quantile	2.65	0.29	A	B			
Automatic Ridler–Calvard	3.11	0.53	A				
Adaptive Median-C	2.06	0.37			C		
Adaptive Mean-C	2.36	0.34		B	C		E
Adaptive Midrange-C	2.62	0.23	A				E
Automatic Otsu	3.10	0.52	A				
Two-dimensional (Otsu)	2.70	0.27	A				E

## Data Availability

The raw data supporting the conclusions of this article will be made available by the authors on request.
